# Metabolic Syndrome Is Associated with Increased Breast Cancer Risk: A Systematic Review with Meta-Analysis

**DOI:** 10.1155/2014/189384

**Published:** 2014-12-29

**Authors:** Ruchi Bhandari, George A. Kelley, Tara A. Hartley, Ian R. H. Rockett

**Affiliations:** ^1^Department of Epidemiology, School of Public Health, West Virginia University, Morgantown, WV 26506, USA; ^2^Department of Biostatistics, School of Public Health, West Virginia University, Morgantown, WV 26506, USA; ^3^National Institute for Occupational Safety and Health, Centers for Disease Control and Prevention, Atlanta, GA 30333, USA

## Abstract

*Background*. Although individual metabolic risk factors are reported to be associated with breast cancer risk, controversy surrounds risk of breast cancer from metabolic syndrome (MS). We report the first systematic review and meta-analysis of the association between MS and breast cancer risk in all adult females.* Methods*. Studies were retrieved by searching four electronic reference databases [PubMed, Cumulative Index to Nursing and Allied Health Literature (CINAHL), Web of Science, and ProQuest through June 30, 2012] and cross-referencing retrieved articles. Eligible for inclusion were longitudinal studies reporting associations between MS and breast cancer risk among females aged 18 years and older. Relative risks and 95% confidence intervals were calculated for each study and pooled using random-effects models. Publication bias was assessed quantitatively (Trim and Fill) and qualitatively (funnel plots). Heterogeneity was examined using *Q* and *I* ^2^ statistics.* Results*. Representing nine independent cohorts and 97,277 adult females, eight studies met the inclusion criteria. A modest, positive association was observed between MS and breast cancer risk (RR: 1.47, 95% CI, 1.15–1.87; *z* = 3.13; *p* = 0.002; *Q* = 26.28, *p* = 0.001; *I* ^2^ = 69.55%). No publication bias was observed.* Conclusions*. MS is associated with increased breast cancer risk in adult women.

## 1. Introduction

Breast cancer, the most common cancer in women worldwide, accounted for 1.7 million new cases in 2012, comprising a quarter of all new cancer cases [1]. While traditional risk factors for breast cancer include age, family history of cancer, and reproductive and menstrual history, the National Cancer Institute also recognizes overweight, lack of physical activity, and consumption of alcohol as risk factors [2]. Several of these risk factors are associated with metabolic syndrome [3].

Metabolic syndrome (MS) is a cluster of pathophysiological disorders comprising central obesity, insulin resistance, high blood pressure, and dyslipidemia. Reaven's definition of MS in 1988 [4] was followed by definitions from the World Health Organization [5], National Cholesterol Education Program's Adult Treatment Panel III (NCEP ATP III) [6], American Heart Association/National Heart, Lung, and Blood Institute [7], and the International Diabetes Federation [8]. The NCEP ATP III guidelines are the most commonly used criteria in the US [6]. These criteria include the presence of three or more of the following [3]: abdominal obesity (waist circumference ≥ 35 inches in women), triglycerides ≥ 150 mg/dL, high density lipoprotein cholesterol (HDL-C) < 50 mg/dL, blood pressure (BP) ≥ 130/85 mmHg, and fasting glucose ≥ 110 mg/dL. MS is estimated to be prevalent in at least a quarter of the adults in the Americas, in Europe, and in India [9].

MS has been identified as a risk factor for several cancers, particularly breast, pancreatic, colorectal, and prostate cancers [10–15]. Individual components of MS, for example, abdominal obesity, high blood glucose, high BP, high triglycerides, and low HDL, are positively associated with the development of certain cancers, most notably breast cancer [16–27]. While studies show a positive association of breast cancer with diabetes [19, 28–33] and obesity [16, 34, 35], others show a negative association with obesity in premenopausal women [36–38]. Mixed results also characterize hypertension [22, 23, 39, 40] and dyslipidemia [22, 41, 42] as risk factors for breast cancer. In addition, although individual components of MS may not be strongly associated with the development of breast cancer, their combination may elevate the risk [13, 14, 43–56]. For example, MS may activate different molecular pathways through endocrine, metabolic, and immune cell changes, which in turn influence breast tumorigenesis [47]. Such pathways that enhance breast cancer cell proliferation and inhibit apoptosis include (1) increased levels of circulating estrogen, for example, estradiol [52, 54, 57], (2) higher levels of insulin [58, 59], (3) decreased level of circulating adiponectin [60], (4) increased plasma leptin concentration [60], and (5) increased production of proinflammatory cytokines, such as interleukin-6 and tumor necrosis factor alpha [61].

Previous epidemiologic studies on MS and breast cancer risk show contrary results. For example, only four [13, 14, 43, 51] of eight studies [13, 14, 43, 48, 51, 62–64] reported a statistically significant association between MS and risk of breast cancer. This might invite a conclusion that the association between MS and breast cancer risk is unknown. However, such an inference would be based on the vote-counting approach, an approach that ignores the magnitude of the association [65].

A recent systematic review and meta-analysis of MS and postmenopausal breast cancer found that MS was moderately associated with the risk of postmenopausal breast cancer [10]. However, to the best of our knowledge, no meta-analytic research has addressed the conflicting results from individual studies of MS and breast cancer risk in all adult women. Therefore, the purpose of this study was to use the aggregate data meta-analytic approach to examine the association between MS and breast cancer risk in women.

## 2. Methods

### 2.1. Study Eligibility

The* a priori* inclusion criteria for this study were as follows: (1) observational studies using cohort (both prospective and retrospective), case-control, or nested case-control study designs; (2) studies examining the association between MS (presence of a cluster of three or more metabolic abnormalities) and breast cancer incidence, as defined by the authors; (3) studies with adult females ≥ 18 years of age as participants; (4) English-language studies published as journal articles, doctoral dissertations, or masters' theses; (5) published and indexed studies up to June 30, 2012; and (6) studies reporting sufficient data (e.g., rate ratios, risk ratios, odds ratios, standardized incidence ratios, hazard ratios, or frequencies) for calculating a common effect size. Neither lobular carcinoma* in situ* nor ductal carcinoma* in situ* breast cancer cases were excluded from the study.

Studies not meeting all inclusion criteria were excluded from this review. Excluded studies were those that (1) were not published as full reports, such as conference abstracts and letters to the editors; (2) only examined individual components of MS; (3) measured the MS variables at time of cancer diagnosis; (4) used cancer mortality, rather than incidence, as the outcome; and (5) were published in a language other than English.

### 2.2. Data Sources

A comprehensive and systematic search was conducted using four electronic databases: PubMed, Cumulative Index to Nursing and Allied Health Literature (CINAHL), Web of Science, and ProQuest (from their commencement to June 30, 2012). Since the term MS dates back to the late 1950s, with variations in use as early as the 1920s, the start dates of each of the databases were used as the commencement date for study search: Web of Science (1900), CINAHL (1952), PubMed (1966), and ProQuest (1861). In addition, cross-referencing from retrieved studies was also performed. Major keywords used in the search for potentially eligible studies included “metabolic syndrome” (“insulin resistance syndrome,” “syndrome x”) and “breast cancer” (“neoplasm and breast”). Using the most recent publication, trials published as duplicate reports (parallel publications) were only included once. All electronic searches were conducted using the graphical user interface for each database. The last search was conducted on June 30, 2012. An initial cut-off point for the inclusion of studies was not used given the difficulty in establishing such a point, as well as our concern about the potential loss of studies that met our eligibility criteria.

### 2.3. Study Selection

At the first screening, one author (RB) screened all abstracts and selected articles for full-text examination. At the second level of the study selection process, two of the authors (RB and TH) examined the full-text articles and then selected the included studies following mutual discussion and consensus.

### 2.4. Data Extraction

Two of the authors (RB and TH) reviewed every study selected and independently extracted data from studies onto electronic coding forms. These forms could hold up to 52 items per study. Attempts were made to contact authors of three of the original studies for missing information [13, 62, 64], but only one provided the requested information [13]. After initial coding, the two coders (RB and TH) reviewed each item for agreement. Discrepancies were resolved by consensus. Using Cohen's kappa (*k*) statistic [66], the overall interrater agreement rate prior to correcting discrepant items was 0.96 for all included studies.

### 2.5. Risk of Bias Assessment

Risk of bias was assessed using a modified version of Strengthening the Reporting of Observational Studies in Epidemiology (STROBE) checklist [67]. The items assessed included (1) study design, (2) adjustments for confounders, (3) selection of participants and their eligibility criteria, (4) measurement of predictor variables, (5) breast cancer diagnosis, (6) study size, (7) handling of missing data, and (8) reasons for nonparticipation of individuals at each stage of the study. A description of the criteria for risk of bias assessment is shown in Table 1. Two of the authors (RB and TH) conducted all assessments, independently of each other. Disagreements were resolved through discussion. No study was excluded based on the results of the risk of bias assessment.

### 2.6. Statistical Analysis

#### 2.6.1. Calculation of Study-Level Effect Sizes

Risk estimates were used to examine the association between MS and risk of breast cancer. These were derived from reported relative risks, odds ratios, hazard ratios, incident rate ratios, or standardized incidence ratios, together with corresponding 95% confidence intervals (CIs), from the original studies. Where necessary and possible, all metrics were converted to risk ratios (RRs). Adjusted risk estimates were pooled for analysis from multivariable models in the original studies. However, for two case-control studies that were included [14, 51], adjusted odds ratios were used because of the lack of the requisite data to convert odds ratios to RRs.

#### 2.6.2. Effect Size Pooling

All RR results were pooled using a random-effects model, an approach that incorporates between-study heterogeneity into the model [68]. A *z*-score two-tailed alpha value ≤ 0.05 was considered to be statistically significant. In addition, 95% CIs were calculated for each result from each study as well as for pooled estimates. Heterogeneity was calculated using the *Q* [69] and *I* ^2^ statistics [70]. An alpha level ≤ 0.10 for the *Q* statistic was considered to be evidence of statistically significant heterogeneity. While somewhat arbitrary, *I* ^2^ values of 25%, 50%, and 75% were considered to represent low, moderate, and high amounts of heterogeneity [70]. Publication bias was assessed using the Trim and Fill approach of Duval and Tweedie [71]. In addition, Rosenthal's Fail-Safe *N* test was used to compute the number of missing null studies that would be needed to nullify the overall pooled RR as being statistically significant [72]. Statistically significant standardized residuals (*p* ≤ 0.05) were considered to be outliers.

#### 2.6.3. Sensitivity Analyses

Influence analysis was conducted with each study result deleted from the model once, in order to examine the effects of each on the overall pooled results. Cumulative meta-analysis, ranked by year, was also conducted in order to examine the accumulation of results over time. A separate pooled analysis, limited to postmenopausal women, was conducted because studies show that MS in postmenopausal women increases the risk of breast cancer [13, 14, 43, 48, 51, 62]. In addition, pooled analyses were conducted with the following caveats* post hoc*: (1) deletion of results from two case-control studies because odds ratios were used instead of RR [14, 51], (2) deletion of results from studies that were not prospective cohort designs [13, 14, 51], and (3) limiting the results to studies that controlled for four or more of the important confounders (as listed in Table 1) [14, 43, 48, 51]. Given the potential for diabetes and diabetes medications to affect breast cancer risk,* post hoc* data analysis was also conducted with studies that included participants with diabetes and/or taking medications for diabetes, deleted from the model [14, 43, 64]. All analyses were performed using Comprehensive Meta-Analysis, Version 2.2 [73].

## 3. Results

### 3.1. Study Characteristics

**Figure 1:**
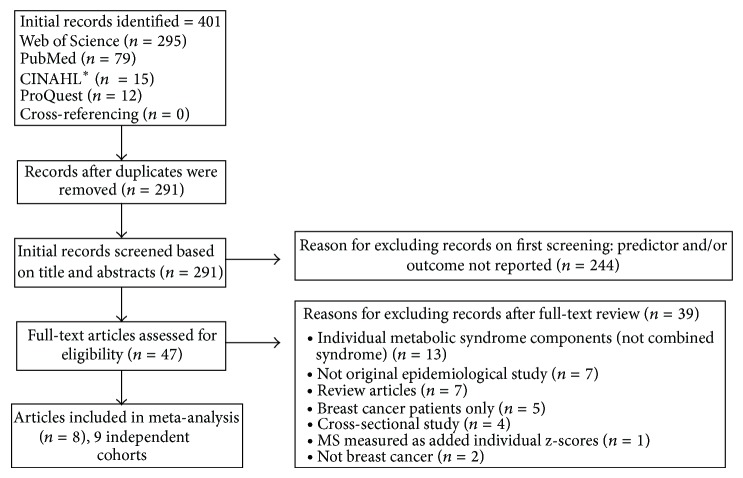
Flow diagram describing the selection of studies. ^*^CINAHL: Cumulative Index to Nursing and Allied Health Literature.

Figure 1 presents a flow diagram of the selection of studies for the meta-analysis. Of the 291 studies screened, 47 (16.2%) were selected for full-text review: 25 from PubMed [14, 43–55, 64, 74–83], 17 from the Web of Science [13, 39, 63, 84–97], one from CINAHL [98], and four from ProQuest [62, 99–101]. Eight (17.0%) of the 47 studies that underwent a full-text review met the eligibility criteria [13, 14, 43, 48, 51, 62–64]. One article [14] presented results for two independent cohorts; thus each cohort was treated independently.

A general description of the included studies is shown in Table 2. Studies were published between 2008 and 2012 and from five different countries. The study designs included four prospective cohorts [48, 62–64], one retrospective cohort [13], one prospective nested case-control study [43], and two case-control studies [14, 51]. The baseline year for cohort inception ranged from 1983 to 2004, with average follow-up ranging between 2.7 and 13.5 years. Sample sizes ranged from 792 to 49,172 (total 97,277) adult females, excluding one study that did not report these data [64]. The ages of the participants ranged from 21 to 86 years. Six studies conducted analyses on postmenopausal women [13, 14, 43, 48, 51, 62]. The results of each cohort or case-control study were initially reported as a hazard ratio [13, 48, 63], incidence rate ratio [43, 62], standardized incidence ratio [64], or odds ratio [14, 51]. Methods for exposure assessment, cancer identification, and control of confounders varied across the eight included studies (Table 3). Seven studies identified the outcome (breast cancer) through histological reports or medical reports or from a cancer registry [13, 14, 43, 48, 51, 62, 64], while one used self-report [63]. Only three studies examined invasive breast cancer cases [43, 48, 64]. One study also reported on the* in situ* breast cancer cases but there were only seven such cases in that study [43]. Another study analyzed all breast cancer cases (*in situ* and invasive) as well as invasive cancers separately, and results remained unchanged [48].

### 3.2. Risk of Bias Assessment

Risk of bias results are shown in Table 4. All of the studies were considered to be at low risk for selection of participants and meeting eligibility criteria in addition to providing adequately powered sample sizes. Out of eight studies, a majority were also considered low risk with respect to study design (six studies) and measurement of the outcome variable (seven studies). In terms of handling potential confounders, half the studies were low risk, three were high risk, and one was unclear risk. Missing confounding variables included education, smoking status, alcohol use, family history of cancer, contraceptive use, or hormonal history. Similarly, half the studies had objective measurements of predictor variables, while the remainder relied on self-report, and were consequently considered high risk. Four studies deleted the participants with missing variables in their analyses (high risk), while two did not report how they handled missing data. Lastly, six studies were considered high risk because they did not report the reasons for nonparticipation of subjects at each stage of follow-up.

### 3.3. Statistical Analysis

#### 3.3.1. Overall Results

**Figure 2:**
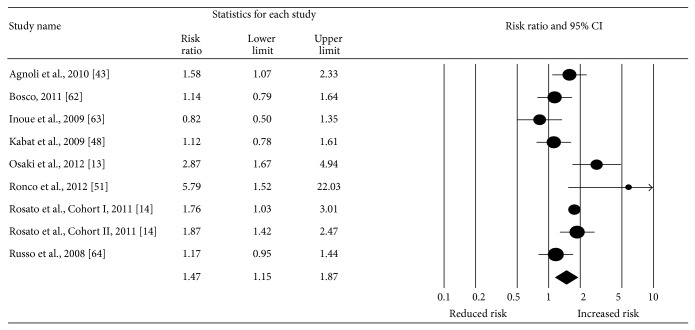
Forest plot for metabolic syndrome and breast cancer risk (random-effects model). The black circles represent the weighted risk ratio (RR) for each result from each study, while the horizontal lines represent the lower and upper 95% confidence intervals (CI) for the RR. The black diamond represents the overall pooled RR, while the left and right sides of the diamond represent the lower and upper 95% CI for the pooled RR. For studies that included more than one definition of metabolic syndrome, the following were used: Agnoli et al. (tertile definition), Bosco (time-independent definition), Osaki et al. (modified NCEP 2001 definition), and Ronco et al. (diabetes, overweight, and hypertension definition).

**Figure 3:**
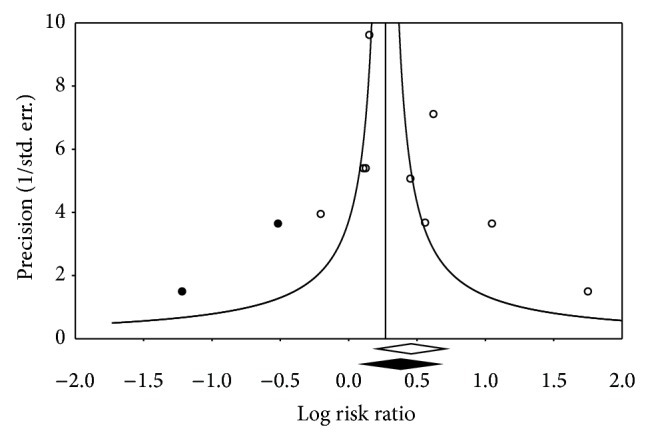
Funnel plot of precision by log risk ratio.

Overall, a statistically significant increase of 47% in the risk for incident breast cancer was observed for adult females with MS (RR: 1.47, 95% CI, 1.15–1.87; *z* = 3.13; *p* < 0.002; *Q* = 26.28, *p* < 0.001; *I* ^2^ = 69.55%) (Figure 2). With the exception of one study [63], all other studies had RR in the direction of increased risk [13, 14, 43, 48, 51, 62, 64]. Funnel plot results for potential publication bias are shown in Figure 3. Using the Trim and Fill approach that resulted in two imputations, the risk decreased by 16% but remained significant (RR: 1.31, 95% CI, 1.01–1.70). The Fail-Safe N was 69, implying that 69 “null” studies would be needed to nullify the statistically significant association between MS and breast cancer risk in adult females. No statistically significant outliers were identified (*p* = 0.06–0.82).

#### 3.3.2. Sensitivity Analyses

**Figure 4:**
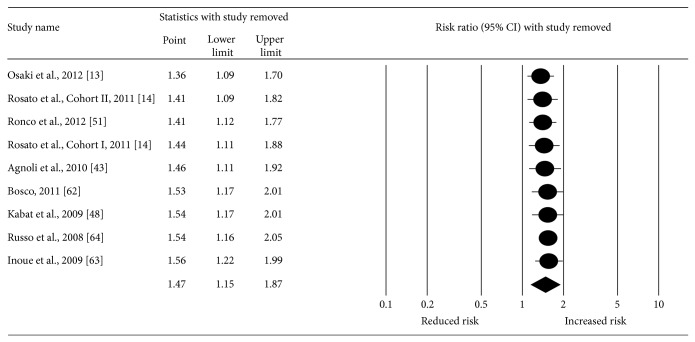
Influence analysis with each result from each study deleted from the random-effects model once. The black circles represent the risk ratio (RR) for each result from each study while the horizontal lines represent the lower and upper 95% confidence interval (CI) for the RR. The black diamond represents the overall pooled result while the left and right sides of the diamond represent the lower and upper 95% CI for the pooled RR. For studies that included more than one definition of metabolic syndrome, the following were used: Agnoli et al. (tertile definition), Bosco (time-independent definition), Osaki et al. (modified NCEP 2001 definition), and Ronco et al. (diabetes, overweight, and hypertension definition).

**Figure 5:**
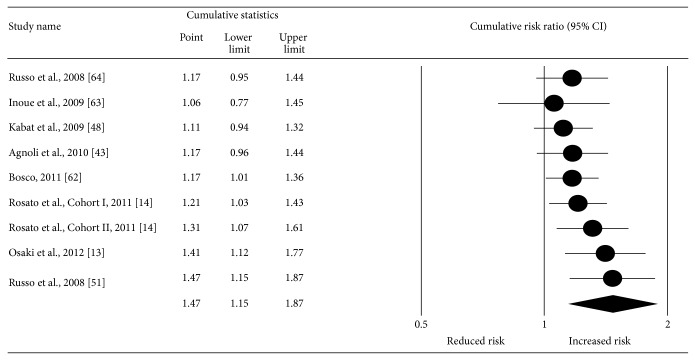
Cumulative meta-analysis, ranked by year and based on a random-effects model. The black circles represent the cumulative risk ratios (RR) while the horizontal lines represent the lower and upper 95% confidence intervals (CI) for the RR. The black diamond represents the overall pooled RR while the left and right sides of the diamond represent the lower and upper 95% CI for the pooled RR. For those studies that included more than one definition of metabolic syndrome, the following were used: Agnoli et al. (tertile definition), Bosco (time-independent definition), Osaki et al. (modified NCEP 2001 definition), and Ronco et al. (diabetes, overweight, and hypertension definition).

With each study deleted from the model once, results remained positive and statistically significant (Figure 4). The pooled RR fell within a range of 20% (RR = 1.36–1.56) and none of the CIs for the point estimates was less than 1.0. Cumulative meta-analysis, ranked by year, revealed that results have been statistically significant since 2011 (Figure 5). Deleting the two case-control studies from the model, the RR for incident breast cancer for women with MS decreased by 18% but was still statistically significant with moderate heterogeneity (RR: 1.29, 95% CI, 1.003–1.67; *z* = 1.98; *p* = 0.05; *Q* = 14.13, *p* = 0.01; *I* ^2^ = 64.61%). When limited to studies with only prospective designs, the RR decreased by 30% but remained statistically significant with very low heterogeneity (RR: 1.17, 95% CI, 1.01–1.36; *z* = 2.04; *p* = 0.04; *Q* = 4.30, *p* = 0.37; *I* ^2^ = 7.04%). When limited to postmenopausal women, breast cancer risk increased by 34% and was still statistically significant with high heterogeneity (RR: 1.81, 95% CI, 1.28–2.56; *z* = 3.37; *p* = 0.001; *Q* = 23.36, *p* = 0.001; *I* ^2^ = 74.32%). When limiting the results to studies that controlled for four or more of the important confounders (as listed in Table 1) [14, 43, 48, 51], breast cancer risk increased by 17% and was statistically significant with moderate heterogeneity (RR: 1.64, 95% CI, 1.23–2.20; *z* = 3.34; *p* = 0.001; *Q* = 8.55, *p* = 0.07; *I* ^2^ = 53.21%). Lastly, when data were analyzed after deleting from the model those studies that had participants with diabetes or taking medications for diabetes [14, 43, 64], the RR was slightly larger than the overall finding but the 95% CI included 1.0 (RR: 1.48, 95% CI, 0.92–2.4; *z* = 1.61; *p* = 0.11; *Q* = 17.4, *p* = 0.02; *I* ^2^ = 76.96%).

## 4. Discussion

The purpose of this aggregate data meta-analysis was to examine the association between MS and the risk for breast cancer in adult females. Overall, the results suggest that there was a modest positive association between MS and risk of breast cancer. This finding is strengthened by the robustness of results from other analyses. These include (1) examination for publication bias, (2) influence analysis with each study being deleted from the model once, (3) deletion of the two case-control studies with odds ratios from the overall model, (4) limiting the analysis to prospective designs, (5) including only postmenopausal women in the analysis, and (6) limiting the results to studies that controlled for four or more of the important confounders. In addition, the results from cumulative meta-analysis, ranked by year, indicate an increasingly statistically significant association since 2011. In contrast, despite a slightly increased mean RR, overlapping CIs were observed when studies that included participants with diabetes or taking medications for diabetes were deleted from the model [14, 43, 64]. However, whether this reduced precision is the result of these specific characteristics or some other factors, for example, loss of power with a reduced number of studies, is not known.

Assessment for risk of bias indicated that a majority of studies were at low risk regarding study design, cancer assessment, and sample size. However, a majority were at high risk or unclear risk in terms of handling of missing data and nonparticipation of subjects at each stage of follow-up. It is suggested that future studies provide complete information on the handling of missing data and on the nonparticipation of subjects at each stage of follow-up.

When limited to postmenopausal women, a stronger association between MS and breast cancer was observed. This association was stronger in case-control and retrospective cohort study designs compared to prospective cohort study designs. These findings concur with those from a recent meta-analysis on MS and breast cancer risk in postmenopausal women [10]. Several studies have shown that MS in this group increases the risk of breast cancer [43, 46, 102], suggesting that the etiology of breast cancer may differ among pre- and postmenopausal women.

There are several potential mechanisms linking MS with an increased risk of breast cancer. First, obese postmenopausal women produce higher levels of estrogens, which in turn increase the biologically available fraction of circulating estradiol by reducing plasma concentration of sex hormone binding globulin (SHBG) [103]. Low plasma SHBG levels are associated with insulin resistance [104, 105] and other components of MS [106, 107]. Second, adipose tissue produces two adipokines (cytokine-like factors), leptin and adiponectin, that affect breast cancer biology [108]. Higher plasma leptin levels are associated with obesity [54, 57, 109], insulin resistance [110, 111], and MS [112, 113]. Leptin stimulates human breast cancer cell lines, whereas adiponectin acts protectively, inhibiting the growth of these cell lines [57, 108, 114]. Obesity is associated with reduced adiponectin levels [115]. Third, insulin has been shown to have a mitogenic effect upon breast cancer cells* in vitro* through several mechanisms [57]. It can act synergistically with estradiol and stimulate proliferation of the cell line [116]. Insulin can also lower SHBG production [117], thereby increasing biologically available estradiol. Moreover, low serum HDL-C concentrations indicate higher circulating bioactive estrogen levels, which in turn may stimulate target breast tissue [77].

The increasing prevalence of MS and its association with breast cancer, among other comorbidities, point toward the critical need to develop public health strategies to manage MS. Given the increasingly large global burden of metabolic risk factors, even a small association with breast cancer can have a substantial public health impact. Risk assessment tools can be developed which incorporate MS as a risk factor for breast cancer. Healthcare providers will then be better equipped to identify high-risk women for primary and secondary prevention.

This study has several strengths. First, to the best of our knowledge, this is the first systematic review and meta-analysis examining the association between MS and risk of breast cancer in all adult women. The analysis incorporates all women and a subanalysis of postmenopausal women. The overlapping meta-analysis on metabolic syndrome and breast cancer was confined to postmenopausal women only [10]. Second, a number of other analyses were performed which strengthened the robustness of findings. Third, the results of this study provide direction for future research on this topic.

This study also has several potential limitations. These include (1) the different methods used to assess exposure, identify cancer, control for confounders, and define MS, (2) limiting studies to those published in English, which may have inflated the results [118], (3) the relatively small number of studies that met the inclusion criteria, (4) the inability of some studies to provide raw data for calculating the RR, (5) the different study designs employed, and (6) the varied populations studied, including those with diabetes and/or taking medications for diabetes. Most notably and with respect to controlling for adiposity, a potential confounder, two of the included studies controlled for BMI [48, 62] but no information was available from the other studies with respect to controlling for BMI or any other obesity-related measures, including such measures of central obesity as waist circumference and waist-to-hip ratio [13, 14, 43, 51, 63, 64]. Given the potential association between breast cancer and adiposity, it may be prudent for future studies to control for this potential confounder. This may be especially true for measures of central adiposity. To this point, Kabat et al. suggested that some, but not all, studies have reported an association between increased central adiposity and an increased risk for postmenopausal breast cancer [48]. Another limitation was a lack of information on tumor subtypes. The inclusion of such information in future studies may be important, given the potential differences in risk according to exposure and disease subtype.

In order to inform and undergird a biological rationale for the observed positive association between MS and breast cancer risk in adult females, future research should comprise analyses based on a standard definition of MS and employ objective and standard biomarkers for assessing each MS component. In addition, adjustments for all important potential confounders need to be made. It would be helpful if future studies examined the relationship between MS and breast cancer risk separately in perimenopausal and premenopausal women since breast cancer in women may be estrogen-independent. Along those lines, not all studies adjusted for hormone replacement therapy, a potential confounder. Future studies should report this information. Furthermore, they need to examine* in situ* and invasive cancers separately in relation to metabolic syndrome. Finally, a focus on obese women with respect to MS and breast cancer seems appropriate.

In conclusion, the overall results of this meta-analysis suggest that there is a modest positive association between MS and risk of breast cancer in adult females.

## Figures and Tables

**Table 1:** Criteria for risk of bias assessment.

Criteria	Low risk	High risk	Unclear risk
Study design	Prospective or retrospective cohort, nested case-control	Case-control	Information not reported

Adjustment of confounders	Adjusted for 4 or more of the following: age, education/income, family history of cancer, hormone therapy use/oral contraceptive use/reproductive history, smoking status, and alcohol consumption	Adjusted for 3 or less of the following: age, education/income, family history of cancer, hormone therapy use/oral contraceptive use/reproductive history, smoking status, and alcohol consumption	Information not reported

Selection of participants and their eligibility criteria	Studies clearly stating their eligibility criteria and the sources and methods of selection of participants	Studies not clearly stating their eligibility criteria and the sources and methods of selection of participants	Information not reported

Measurement of predictor variables	Identified through objective measures	Self-reported or pharmaceutical prescriptions	Information not reported

Breast cancer diagnosis	Histologically confirmed or identified through cancer registry/medical records	Self-reported	Information not reported

Study size	Large enough for adequate power	Not large enough for adequate power	Information not reported

Handling of missing data	Missing data analysis specified	Missing data deleted from analysis	Information not reported

Reasons for nonparticipation of individuals at each stage of the study	Reasons clearly reported for each stage of study	Reasons not reported for each stage of study	Information not reported

**Table 2:** Characteristics of studies.

Author	Year	Country	Study design	Sample size	Baseline year	Follow-up years	Age	Breast cancer cases	Menopausal status	Statistic
Agnoli et al. [43]	2010	Italy	Prospective nested case-control	792	1987–92	2003	35–69	163	Post	Rate ratios
Bosco [62]	2011	USA	Prospective cohort	49,172	1995	2007	21–69	1228	Mixed, post	Incidence rate ratios
Inoue et al. [63]	2009	Japan	Prospective cohort	18,176	1990–94	2004	40–69	120	Mixed, post	Hazard ratios
Kabat et al. [48]	2009	USA	Prospective cohort	4,888	1993–98	2005	50–79	165	Post	Hazard ratios
Osaki et al. [13]	2012	Japan	Retrospective cohort	15,386	1992–2000	2007	20+	77	Mixed, post	Hazard ratios
Ronco et al. [51]	2012	Uruguay	Case-control	912	2004	2009	<70	367	Post	Odds ratios
Rosato et al.—Cohort I [14]	2011	Italy	Case-control	3,858	1983	1994	33–86	1,988	Post	Odds ratios
Rosato et al.—Cohort II [14]	2011	Italy and Switzerland	Case-control	4,093	1991	2007	33–79	1,881	Post	Odds ratios
Russo et al. [64]	2008	Italy	Prospective cohort	Not reported	1999	2005	≥40	99	Mixed	Standardized incidence ratios

Author	Exposure assessment			Cancer identification			Confounders			

Agnoli et al. [43]	Questionnaire, anthropometric measures, and fasting blood draw			Cancer registry			Age, age at menarche, age at first birth, years from menopause, number of full-term pregnancies, oral contraceptives, hormone therapy, education, cancer in first degree relatives, breastfeeding, smoking, and alcohol consumption			
Bosco [62]	Questionnaire			Medical records or cancer registry data			Age, education, BMI at 18, and vigorous activity			
Inoue et al. [63]	Questionnaire, anthropometric measures, and fasting and nonfasting blood draw			Self-report			Age, study area, smoking status, ethanol intake, physical activity, and total cholesterol			
Kabat et al. [48]	Questionnaire, anthropometric measures, and fasting blood draw			Self-report confirmed by medical records and tumor registry abstracts			Age, education, ethnicity, BMI, oral contraceptive use, postmenopausal hormone therapy, age at menarche, age at first birth, age at menopause, alcohol, family history of breast cancer, history of breast biopsy, physical activity, energy intake, and smoking status			
Osaki et al. [13]	Questionnaire, anthropometric measures, and fasting blood draw			Cancer registry			Age, smoking, and heavy drinking			
Ronco et al. [51]	Questionnaire and anthropometric measures after cancer			Histologically confirmed breast cancer			Age, residence, age at menarche, parity, age at first live birth, months of breastfeeding, use of oral contraceptives, BMI, menopausal status, family history of breast cancer, and intake of beef, tomatoes, and oranges			
Rosato et al. [14]	Questionnaire and waist circumference measure			Histologically confirmed breast cancer			Age, study center, study period, education, alcohol consumption, age at menarche, parity and age at first birth, age at menopause, hormone replacement therapy use, and family history of breast cancer			
Russo et al. [64]	Pharmaceutical prescriptions for MS			Cancer registry			Not reported			

*Notes*. BMI: body mass index.

**Table 3:** Definitions and criteria for metabolic syndrome in the included studies.

Agnoli et al. [43]	2 definitions (≥3 of the following components). (1) The highest or lowest (HDL-C) tertiles in controls: WC > 86 cm; triglycerides > 126 mg/dL; HDL-C ≤= 55 mg/dL; fasting glucose > 88 mg/dL (or previously diagnosed Type 2 Diabetes Mellitus); mean BP ≥ 106.5 mmHg (or treatment for previously diagnosed HTN). (2) NCEP: WC > 88 cm; triglycerides ≥ 150 mg/dL; HDL-C < 50 mg/dL; SBP ≥ 130 mmHg or diastolic BP ≥ 85 mmHg; fasting glucose ≥ 110 mg/dL.

Bosco [62]	≥3 of the following components: WC ≥ 88 cm; Type 2 Diabetes Mellitus self-reported diagnosis at ≥ 30 years at baseline; HTN self-reported diagnosis plus diuretics or hypertensive medication use at baseline; cholesterol self-reported diagnosis of high cholesterol and cholesterol-lowering medication at baseline.

Inoue et al. [63]	2 definitions. (1) Grundy (NHLBI 2005): any 3 or more: BMI ≥ 25 kg/m^2^; HTN ≥ 130/85 mmHg or medication use; glucose ≥ 100 mg/dL fasting or 140 mg/dL nonfasting or on treatment; low HDL-C < 50 mg/dL; triglycerides ≥ 150 mg/dL. (2) IDF: overweight and at least 2 other components.

Kabat et al. [48]	ATP III modified to exclude those with glucose ≥ 126 mg/dL or those taking diabetic medication.

Osaki et al. [13]	6 definitions: Japan 2005, modified NCEP 2001, modified NCEP 2004, modified IDF 2006, modified WHO 1999, and NCEP 2001 with BP 140/90.

Ronco et al. [51]	2 definitions. (1) Diabetes + overweight + HTN. (2) Diabetes + overweight + dyslipidemia.

Rosato et al. [14]	Combined presence of diabetes, drug-treated HTN, drug-treated hyperlipidemia (as a proxy indicator of elevated triglycerides and reduced HDL-C), and WC ≥ 88 cm or BMI ≥ 30 kg/m^2^ when WC was missing.

Russo et al. [64]	Pharmacological definition: patients who chronically received antihypertensive, glucose-lowering, and lipid modifying drugs.

*Notes.* BMI: body mass index; BP: blood pressure; HDL-C: high density lipoprotein cholesterol; HTN: hypertension; WC: waist circumference; IDF: International Diabetes Federation; NCEP ATP III: National Cholesterol Education Program's Adult Treatment Panel III; NHLBI: National Heart, Lung, and Blood Institute; WHO: World Health Organization.

**Table 4:** Study-level results for risk of bias assessment.

	Agnoli et al. [43]	Bosco [62]	Inoue et al. [63]	Kabat et al. [48]	Osaki et al. [13]	Ronco et al. [51]	Rosato et al. [14]	Russo et al. [64]
Methods								
Study design	Low	Low	Low	Low	Low	High	High	Low
Variables (confounders)	Low	High	High	Low	High	Low	Low	Unclear
Participants (eligibility, selection)	Low	Low	Low	Low	Low	Low	Low	Low
Data sources/predictor measurement	Low	High	Low	Low	Low	High	High	High
Data sources/outcome measurement	Low	Low	High	Low	Low	Low	Low	Low
Study size (adequate power)	Low	Low	Low	Low	Low	Low	Low	Low
Missing data analysis	High	High	High	Low	High	Low	Unclear	Unclear
Results								
Participants (non-participation)	High	High	High	High	Low	Low	High	High
